# Evaluation of putative CSF biomarkers in paediatric spinal muscular atrophy (SMA) patients before and during treatment with nusinersen

**DOI:** 10.1111/jcmm.16802

**Published:** 2021-07-27

**Authors:** Jessika Johannsen, Deike Weiss, Anne Daubmann, Leonie Schmitz, Jonas Denecke

**Affiliations:** ^1^ Department of Pediatrics University Medical Center Hamburg‐Eppendorf Hamburg Germany; ^2^ Medical Biometry and Epidemiology University Medical Center Hamburg‐Eppendorf Hamburg Germany

**Keywords:** antisense, cerebrospinal fluid, motor function scores, neurodegeneration, oligonucleotide

## Abstract

Spinal muscular atrophy (SMA) is a genetic neurodegenerative disorder leading to immobilization and premature death. Currently, three alternative therapeutic options are available. Therefore, biomarkers that might reflect or predict the clinical course of the individual patient with treatment are of great potential use. Currently, the antisense oligonucleotide nusinersen is the prevalent and longest validated therapy for SMA. We analysed CSF candidate biomarkers for degenerative CNS processes (namely phosphorylated heavy chain (pNf‐H), light‐chain neurofilaments (NfL), total tau protein (T‐Tau), neurogranin, β‐secretase BACE‐1 and alpha‐synuclein) in 193 CSF samples of 44 paediatric SMA types 1, 2 and 3 patients before and under nusinersen treatment and related them to standardized clinical outcome scores in a single‐centre pilot study. pNf‐H and NfL correlated with disease severity and activity, emphasizing their relevance as marker of neuronal loss and clinical outcome. T‐Tau was significantly correlated with motor function scores in SMA type 1 making it an interesting marker for treatment response. Additionally, baseline T‐Tau levels were elevated in most SMA patients possibly reflecting the extension of neuronal degeneration in paediatric‐onset SMA. Further investigations of these CSF proteins might be beneficial for paediatric SMA subtypes and treatment modalities as an indicator for clinical outcome and should be analysed in larger cohorts.

## 1 INTRODUCTION

In recent years, new therapies emerge in an increasing number of rare diseases and specific biomarkers reflecting the disease course and its progression become increasingly important. In 2017, the antisense oligonucleotide nusinersen was approved for the intrathecal treatment of all types of 5q‐associated spinal muscular atrophy (SMA) in Europe.^1^ SMA is an autosomal recessive neuromuscular disease caused by homozygous deletion or mutation of the *SMN1* (survival motor neuron) gene. Lack of SMN protein mainly leads to dysfunction and later apoptosis of the lower motor neuron resulting in progressive muscular weakness, respiratory insufficiency and bulbar symptoms. However, the *SMN1* gene is ubiquitously expressed and the relevance of SMN protein in other tissues is not fully understood.^2^ The *SMN2* gene as a nearly identical copy of *SMN1* only produces about 10% of full‐length SMN protein due to altered splicing.^3^ The copy number of the *SMN2* gene shows remarkable interindividual differences with later onset and milder course (SMA types 1–4) in SMA patients with more *SMN2* copies.^4^ Neuropathological findings in deceased patients with SMA type 1, the most severe SMA subtype, showed not only neuronal degeneration in lower motor neuron, but also in the cerebral cortex, basal ganglia, brain stem and cerebellum.^5^

Nusinersen alters the splicing of SMN2 pre‐mRNA increasing the concentration of functional SMN protein. Clinical trials have shown promising clinical efficacy in paediatric SMA patients compared to untreated patients.^6^, ^7^, ^8^ To date, individual clinical improvement cannot be predicted by specific clinical or biochemical markers. The presence of biomarkers correlating with the efficacy of treatment or even predicting outcome or clinical success of a therapy is essential considering alternative therapeutic options, for example gene replacement therapy.^9^

Until now, neurodegeneration in various diseases has been addressed by different biomarkers in plasma and/or cerebrospinal fluid (CSF) such as neurofilaments and Tau protein in neuronal injury or loss, neurogranin in synaptic loss and β‐secretase BACE‐1 in hypomyelination.^10^, ^11^, ^12^, ^13^ Furthermore, the accumulation of alpha‐synuclein has been associated with the pathomechanisms in different neurodegenerative diseases (ie Parkinson's disease, multiple system atrophy).^13^ In order to better understand neurodegeneration in SMA, some efforts to characterize the disease at baseline in clinical and biochemical aspects have been made: Kolb et al. found altered electrophysiological and plasma protein parameters and lower SMN mRNA levels in SMA patients than in the control group. However, the course of these parameters during disease progression was not determined.^14^ Recently, elevated plasma pNf‐H levels were correlated with disease severity and inversely correlated with the age at presentation in 117 nusinersen‐treated SMA infants included in the ENDEAR study (NCT02193074).^15^ During nusinersen treatment, pNf‐H decreased faster and to lower values in the plasma compared to sham control–treated infants.^15^ However, data of baseline CSF pNf‐H levels, CSF NfL levels and their course during nusinersen treatment in paediatric SMA patients are still limited.^16^ As the investigation of neurofilaments only addresses one aspect of neurodegeneration, the course of additional biomarkers before and during nusinersen treatment might be able to further characterize the mechanisms of damage in SMA and to predict clinical outcome under treatment. Therefore, we investigated a number of candidate biomarkers in CSF samples of patients with SMA types 1–3 at baseline and under treatment with nusinersen and aimed to find a specific profile of these proteins under treatment. The aim of the study was to answer the questions 1. whether there are differences between the SMA subtypes, 2. whether nusinersen treatment affects the candidate biomarkers levels, 3. whether there is a correlation between clinical scores and candidate biomarkers levels and 4. whether candidate biomarkers levels predict clinical response.

## 2 METHODS

### 2.1 Patients and Study Samples

In the Department of Pediatrics of the University Medical Center Hamburg‐Eppendorf, treatment with nusinersen was initiated within the early access programme (EAP) in January 2017 for patients with SMA type 1 Patients with SMA types 2 and 3 have been included since July 2017 after the medication was approved by the European Medicines Agency (EMA). All patients had a documented 5q‐associated SMA. Written informed consent from the parents or guardians were obtained for the lumbar puncture; the intrathecal administration of nusinersen and the storage of CSF samples followed institutional guidelines. The biomarker investigation in CSF samples was approved by the local ethics committee (PV5865), and parents or guardians and children who were able to read provided written informed consent.

In accordance with the recommended dosing schedule, the intrathecal administration of nusinersen was performed on treatment days 0, 14, 28 and 63 (loading dosing) followed by maintenance doses every 4 months. CSF samples were obtained at the lumbar punctures necessary for nusinersen application. Only patients with baseline CSF samples before the first nusinersen administration (day 0), and CSF samples without pleocytosis and/or haemoglobin were included in analyses. CSF samples were immediately stored and transported at −80℃.

Motor function analysis using CHOP INTEND (Children's Hospital of Philadelphia Infant Test of Neuromuscular Disorders) in SMA type 1 patients and both HFMSE (Hammersmith Functional Motor Scale Expanded) and RULM (revised upper limb module) in SMA type 2 and 3 patients were performed on treatment day 0, treatment day 63 and then every 4 months with the next nusinersen application.

The interval between (reported) symptom onset and first nusinersen treatment (=disease duration) was collected.

### 2.2 Laboratory Analysis

The CSF samples were analysed with ELISAs commercialized by EUROIMMUN AG (Lübeck, Germany): BACE‐1 ELISA (EQ 6541–9601‐L), Total‐Tau ELISA (EQ 6531–9601‐L), Alpha‐Synuclein ELISA (EQ 6545–9601‐L), Neurogranin (trunc P75) ELISA (EQ 6551–9601‐L) and Neurofilament heavy (pNf‐H) ELISA (EQ 6561–9601) were used according to the manufacturer's test instruction. The Neurofilament light (NfL) was still a prototype ELISA developed by EUROIMMUN, which is not commercially available yet.

### 2.3 Statistical Analysis

Sociodemographic variables were reported with mean and standard deviation as well as absolute and relative frequencies depending on the scale level of the variables.

For determining the course of the motor function and the CSF candidate biomarkers, we used a linear mixed model in each case with the changes from baseline as the dependent variable, time point, age (in respect to candidate biomarkers), SMA type, baseline value of the respective dependent variable and interaction between time point and SMA type as fixed effects and patient as random effect. If the interaction was not significant, we eliminated it from the model. That was the case for neurogranin, β‐secretase BACE‐1 and alpha‐synuclein, HFMSE and RULM. To assess the association between motor function and the biomarkers, we used a linear mixed model with motor function as dependent variable, biomarkers as fixed effect and patient as random effect. For every combination, we conducted a single model. To assess the association between the changes from baseline of motor function and the changes from baseline of biomarker levels, we calculated a linear regression in each case with motor function on day 180 and day 300, respectively, as the dependent variable, SMA type, baseline value of the respective dependent variable, changes from baseline of the preceding times and the interaction between SMA type and changes from baseline of the preceding times as fixed effects. Including an age adjustment in the model was not possible due to insufficient sample size. To evaluate the association between the changes from baseline of motor function and the biomarker levels at baseline, we calculated a linear mixed model with changes from baseline of motor function as the dependent variable, SMA type (in case of RULM and HFMSE), time point, baseline value of the respective dependent variable and the biomarker level at baseline as fixed effects and patient as random effect. To assess the association between motor function at baseline respectively biomarkers at baseline, and the duration of the disease, we evaluated a linear model with motor function, and biomarker, respectively, as dependent variable, SMA type, duration and the interaction between both variables as fixed effects. The model used for the biomarker included age as fixed effect. To assess the association between the changes from baseline of biomarker levels and the duration of the disease, we calculated a linear mixed model with changes from baseline as the dependent variable, SMA type, time point, duration, the baseline value of the respective biomarker and the interaction between SMA type and duration as fixed effects and patient as random effect. In models with CHOP INTEND as dependent variable, we omitted SMA type and possible interactions. Due to the exploratory character of our study, we did not correct for multiplicity. For all tests (each with a two‐sided hypothesis), level of significance was set at *p *< 0.05. All analyses were conducted with SAS, version 9.4.

## 3 RESULTS

### 3.1 Patients and Study Samples

In total, 193 CSF samples of 44 5q‐associated SMA patients were obtained. Sixteen patients with SMA type 1 (male 5, female 11, average age at treatment start: 16.0 months (range: 2–68 months)), 16 patients with SMA type 2 (male 5, female 11, average age at treatment start: 78.8 months (range 21–152 months)) and 12 patients with SMA type 3 (male 5, female 7, average age at treatment start: 119.9 months (range 38–208 months)) were initially included. Finally, 15 patients with SMA type 1, 15 patients with SMA type 2 and 10 patients with SMA type 3 were included in the statistical analysis determining the course of CSF proteins during nusinersen treatment. Samples of 1 SMA type 1 patient, 1 SMA type 2 patient and 2 girls with SMA type 3 had to be excluded because of a missing baseline (ie before treatment) sample. Only patients with a valid baseline value and at least one valid follow‐up value in our analysis population were included. Further, 7 samples had to be excluded from analyses because of blood contamination.

The initial nusinersen dose was applied in patients between the age of 9.0 weeks and 17 years and 8 weeks.

Clinical and demographic data are shown in Table 1.

**TABLE 1:** Clinical and demographic characteristics of the study population

	SMA type 1	SMA type 2	SMA type 3
Total number of patients	16	16	12
Number of patients used for statistical analysis	15	15	10
SMN2 copy number			
2	13	1	1
3	3	10	1
4	0	3	7
Unknown		2	1
Mean age at treatment start (in months)	16.0	78.8	119.9
Age range at treatment start (in months)	2‐68	21‐152	38‐208
Mean disease duration in months (range)	13.8 (0.5‐63)	68.8 (6‐146)	79.6 (5‐171)
Female gender	11	11	7
CSF samples excluded due to missing baseline samples	1	1	2
CSF samples excluded due to blood contamination	4	3	0

### 3.2 Motor function assessment

**FIGURE 1:**
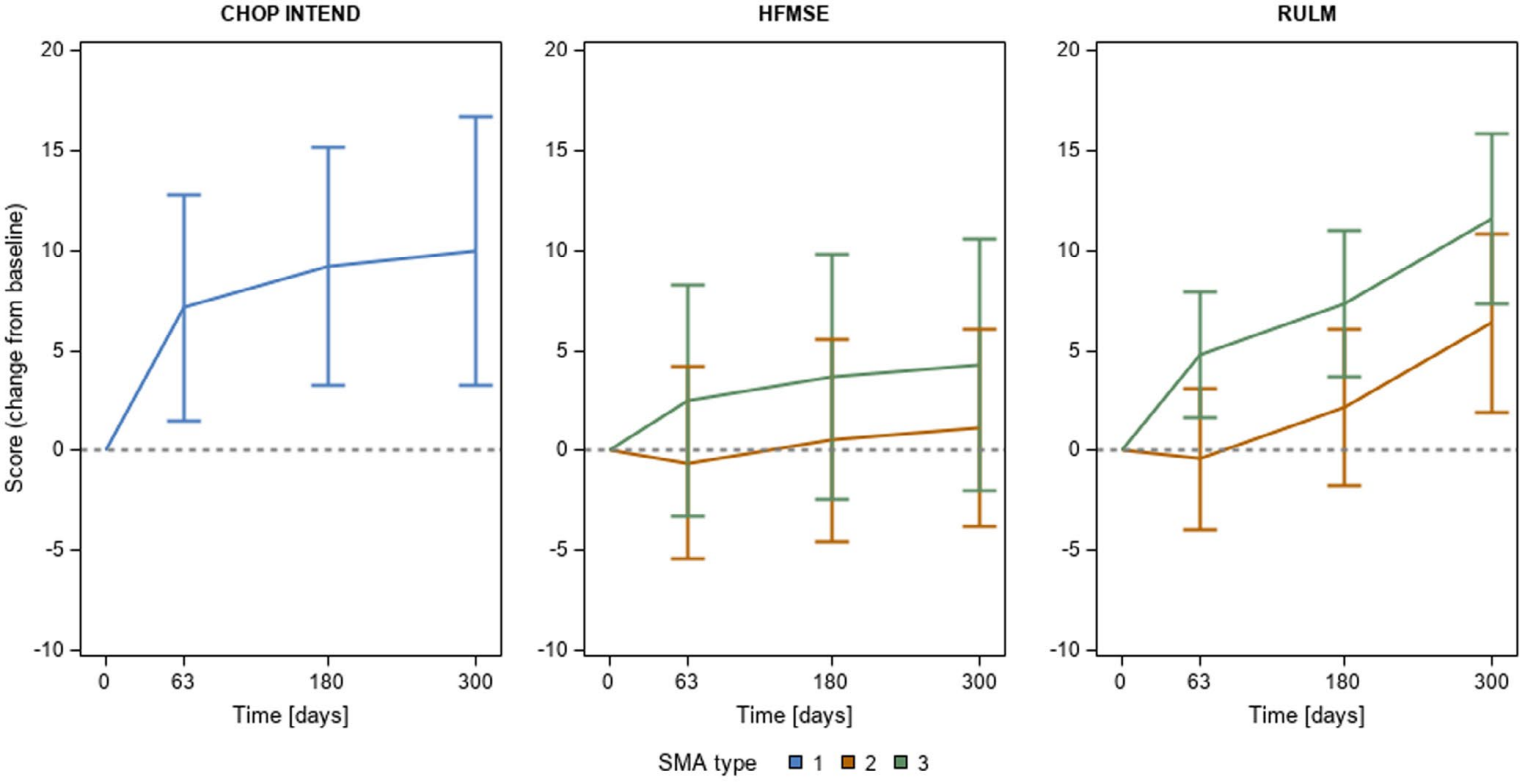
The course of motor function scores before (baseline) and with nusinersen treatment in SMA patients measured by CHOP INTEND, HFMSE and RULM. Time points are shown on x‐axis and changes of scores from baseline on y‐axis. SMA types are presented separately with different colours (blue: type 1, orange: type 2; green: type 3)

Motor function scores at baseline and their course during treatment are shown in Figure 1 and as mean total values and as values reflecting the change from baseline in Table 2a. Number of patients assessed by CHOP INTEND, HFMSE and RULM, respectively, are included in Table 2a. SMA type 1 patients showed a significant gain of motor function reflected by increasing CHOP INTEND scores during treatment. In SMA type 2 and 3 patients, motor function scores improved as well during treatment, but only upper limb function (measured by RULM) was significantly different between baseline and day 300 of treatment.

**TABLE 2a:** Mean motor function scores and changes from baseline in CHOP INTEND, HMFSE and RULM at baseline and day 63, 180 and 300 of nusinersen treatment, separately shown for each SMA type. P values <0.05 are shown in bold

	SMA type 1							SMA type 2									SMA type 3						
				Change from baseline								Change from baseline								Change from baseline			
Outcome variables	N	Mean	SD	Adjusted Mean	95% CI		p Value		N	Mean	SD	Adjusted Mean	95% CI		p Value	N		Mean	SD	Adjusted Mean	95% CI		p Value
CHOP INTEND								HFMSE															
Baseline	16	18.6	13.1					Baseline	12	7.5	4.9					11		46.8	14.7				
63 days	12	24.8	11.4	7.1	1.5	12.8	**0.017**	63 days	9	10.3	12.4	−0.6	−5.4	4.2	0.774	10		47.2	15.1	2.5	−3.3	8.3	0.357
180 days	10	29.1	14.9	9.2	3.3	15.2	**0.005**	180 days	9	9.6	8.0	0.5	−4.5	5.6	0.819	3		41.0	5.2	3.7	−2.4	9.7	0.209
300 days	7	31.4	21.6	9.9	3.2	16.7	**0.007**	300 days	8	11.1	7.7	1.2	−3.8	6.1	0.610	2		52.0	8.5	4.3	−1.9	10.6	0.159
																						
								RULM															
								Baseline	7	9.0	6.4					10		30.4	7.2				
								63 days	8	12.5	5.9	−0.4	−3.9	3.1	0.780	10		32.3	5.9	4.8	1.6	7.9	**0.009**
								180 days	9	13.4	3.9	2.2	−1.8	6.1	0.235	3		32.3	5.0	7.4	3.7	11.0	**0.002**
								300 days	8	15.3	7.3	6.4	1.9	10.8	**0.012**	2		33.0	4.2	11.6	7.3	15.9	**<0.001**

### 3.3 Correlation of biomarkers with motor function scores

**FIGURE 2:**
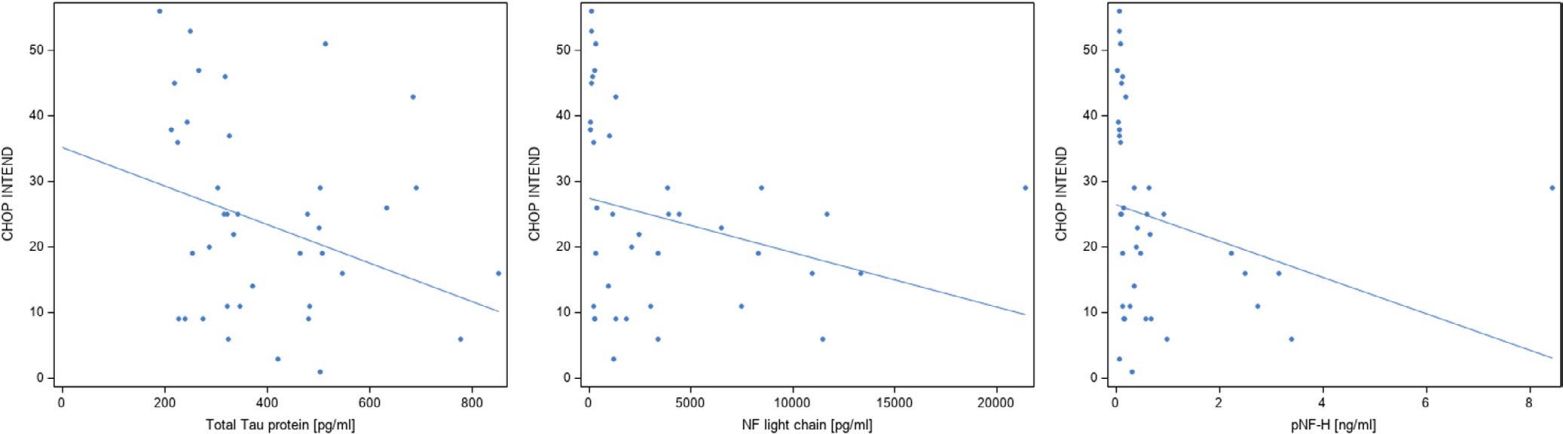
Correlation of T‐Tau, NfL, p‐NfH levels and motor function scores, measured by CHOP INTEND in SMA type 1 patients. CSF protein concentrations are shown on x‐axis and motor function scores on y‐axis

Since CHOP INTEND was performed in SMA type 1 patients and RULM and HFMSE was performed in SMA types 2 and 3, correlation of biomarkers and motor function scores had to be evaluated separately for type 1 and type 2/3, respectively. Results are shown in Table 2b. A significant inverse correlation between CHOP INTEND scores and Tau and pNf‐H concentration could be found in SMA type 1 after treatment with nusinersen (Figure 2). In SMA type 2 and 3 patients, there was a significant correlation of RULM scores and Neurogranin concentration as well as a significant inverse correlation of pNf‐H‐concentration with RULM and HFMSE scores and of NfL and HFMSE scores.

**TABLE 2b:** Correlation between motor function, measured by CHOP INTEND in SMA type 1 patients and RULM and HFMSE, respectively, in SMA type 2/3 patients and concentration of CSF protein. §: Example: If T‐Tau is 1000 times higher, the CHOP INTEND score is on average 29.5 times lower. P values <0.05 are shown in bold

CSF proteins	CHOP INTEND SMA type 1				RULM SMA type 2 and 3				HFMSE SMA type 2 and 3			
	Slope	95% CI		P Value	Slope	95% CI		p Value	Slope	95% CI		p Value
BACE−1 (1000 Units)	2.6	−9.19	14.31	0.659	4.3	−2.71	11.25	0.219	1.1	−5.53	7.81	0.729
T‐Tau (1000 Units)^§^	−29.5	−53.36	−5.65	**0.017**	10.3	−13.67	34.34	0.384	1.0	−20.25	22.24	0.924
α‐Synuclein (1000 Units)	3.1	−4.49	10.72	0.408	2.1	−0.56	4.76	0.116	0.4	−1.36	2.17	0.643
Neurogranin (1000 Units)	15.3	−6.72	37.37	0.166	18.8	5.65	31.95	**0.007**	11.2	−2.86	25.21	0.114
NfL (1000 Units)	−0.8	−1.67	0.00	0.051	−6.7	−15.02	1.70	0.114	−7.5	−14.24	−0.77	**0.030**
pNf‐H (1 Units)	−2.8	−5.52	−0.02	**0.048**	−10.5	−20.55	−0.35	**0.043**	−11.1	−19.07	−3.18	**0.008**

### 3.4 Evaluating the predictive power of biomarkers for the clinical outcome

Correlation between change in biomarker levels from baseline to treatment day 63 and 180, respectively, and change of motor function parameters from baseline to day 180 and 300 of treatment were calculated in order to evaluate an early predictive impact of the biomarkers or changes of biomarkers on the prospective clinical therapeutic response. Only in SMA type 1 patients, a change of both BACE‐1 and alpha‐synuclein levels at day 180 significantly correlated with a change in the CHOP INTEND score at treatment day 300 (*p *= 0.040 and *p *= 0.013, respectively). No significant correlation between the biomarker at baseline and change of motor function scores was found (data not shown).

### 3.5 Correlation between disease duration and CSF candidate biomarkers

Only in SMA type 1 patients, longer disease duration significantly correlated with lower baseline levels of T‐Tau, NfL and p‐NfH and lower changes of NfL levels from baseline to treatment day 63, 180 and 300. Additionally, longer disease duration was correlated with lower changes of alpha‐synuclein concentrations from baseline to treatment day 63, 180 and 300 (*p *= 0.003). With age adjustment to decrease ageing effects on the correlation, the effect was still significant for the baseline levels of NfL in SMA type 1 (Table 3).

**TABLE 3:** Correlation between 1. disease duration and concentration of CSF proteins at baseline and 2. disease duration and concentration changes of CSF proteins from baseline to treatment day 63, 180 and 300, respectively, separately reported for each SMA type. §: Example: If disease duration is one month longer, the baseline concentration of T‐Tau is on average 4.16 units lower. #: With age adjustment, p values were not significant anymore. P values <0.05 are shown in bold

CSF proteins	SMA type	Correlation between disease duration and CSF proteins concentration at baseline				Correlation between disease duration and concentrations changes of CSF proteins from baseline to treatment day 63, 180, 300			
		Slope	95% CI		P value	Slope	95% CI		P value
BACE−1 (1000 Units)	1	6.4	−5.58	18.38	0.285	−3.2	−8.75	2.42	0.258
	2	−1.1	−6.32	4.05	0.658	−1.0	−3.53	1.47	0.410
	3	1.2	−3.27	5.69	0.588	−0.8	−2.95	1.30	0.439
T‐Tau (1000 Units)	1	−4.2 ^§^	−8.10	−0.22	**0.039 ^#^**	−0.5	−2.81	1.83	0.672
	2	−1.3	−2.99	0.42	0.135	0.1	−0.91	1.14	0.815
	3	−0.8	−2.26	0.69	0.288	−0.6	−1.46	0.24	0.156
α‐Synuclein (1000 Units)	1	0.9	−23.71	25.60	0.938	−9.5	−26.29	7.38	0.262
	2	3.9	−6.70	14.63	0.455	−11.2	−18.37	−4.05	**0.003**
	3	−3.1	−12.31	6.14	0.501	−0.6	−6.69	5.42	0.834
Neurogranin (1000 Units)	1	0.2	−6.08	6.48	0.949	−1.8	−3.78	0.19	0.075
	2	−1.7	−4.40	1.04	0.217	−0.6	−1.53	0.34	0.209
	3	−1.9	−4.25	0.44	0.109	−0.6	−1.36	0.24	0.163
NfL (1000 Units)	1	−174.0	−271.86	−76.14	**0.001**	−45.5	−84.98	−6.11	**0.025**
	2	−0.2	−42.51	42.18	0.994	0.4	−15.21	15.92	0.963
	3	2.1	−34.47	38.74	0.906	1.2	−12.14	14.50	0.859
pNf‐H (1 Units)	1	−0.05	−0.0874	−0.0140	**0.008 ^#^**	−0.001	−0.0045	0.0025	0.572
	2	0.0002	−0.0157	0.0160	0.984	0.0001	−0.0013	0.0015	0.912
	3	0.0005	−0.0132	0.0143	0.938	0.001	−0.0002	0.0022	0.096

### 3.6 CSF candidate biomarkers

**FIGURE 3:**
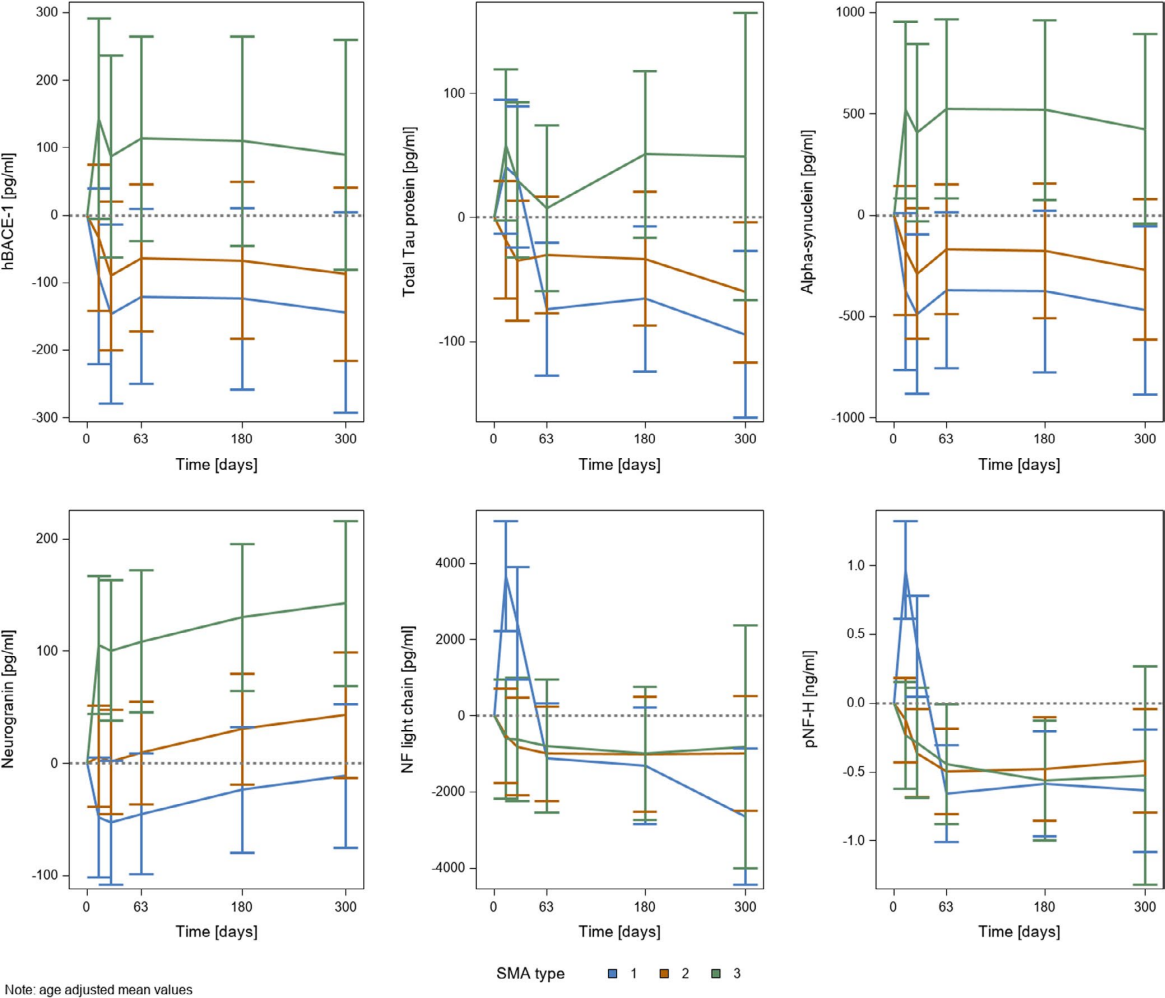
Age‐adjusted mean concentration changes from baseline of CSF BACE‐1, total Tau protein, alpha‐synuclein, neurogranin, NfL, pNf‐H before and with nusinersen treatment, separately shown for each SMA type (blue: type 1, orange: type 2, green: type 3). Time points are shown on x‐axis and changes of protein levels from baseline values on y‐axis

The mean concentrations of putative CSF biomarkers in all statistically analysed samples and the adjusted mean of the concentration differences compared to baseline levels are reported in Table 4 and Figure 3, respectively.

**TABLE 4:** Mean concentrations of CSF proteins at baseline and at treatment day 63, day 180 and day 300, separately reported for each SMA type. P values <0.05 are shown in bold

Outcome variables	SMA type 1				SMA type 2				SMA type 3			
	N	Mean	SD	p Value	N	Mean	SD	p Value	N	Mean	SD	p Value
BACE−1 (pg/ml)												
Baseline	15	940.67	491.64		15	1154.57	276.37		10	1470.14	434.98	
Day 63	14	834.48	395.42	0.069	15	1085.91	380.55	0.251	10	1392.26	483.51	0.142
Day 180	11	961.97	547.67	0.070	10	1045.18	358.33	0.257	10	1548.12	401.74	0.164
Day 300	8	928.84	492.38	0.056	10	959.08	407.80	0.180	3	1467.48	296.06	0.299
T‐TAU (pg/ml)												
Baseline	15	465.27	182.06		15	339.28	98.27		10	341.46	144.00	
Day 63	14	352.38	123.64	**0.008**	15	312.37	109.95	0.208	10	329.04	115.49	0.819
Day 180	11	359.44	169.43	**0.029**	10	327.01	110.02	0.221	10	379.33	106.35	0.134
Day 300	8	359.97	130.74	**0.007**	10	289.17	114.65	**0.036**	3	373.23	11.61	0.401
α‐Synuclein (pg/ml)												
Baseline	15	1603.28	696.87		15	2170.66	977.14		10	2284.78	777.29	
Day 63	14	1616.35	601.35	0.060	14	1790.77	910.63	0.296	10	2410.91	689.63	**0.020**
Day 180	10	1758.04	915.06	0.064	9	1803.97	687.13	0.299	10	2317.54	1184.03	**0.022**
Day 300	8	1717.38	796.72	**0.026**	10	1638.93	702.36	0.127	3	2009.26	59.25	0.073
Neurogranin (pg/ml)												
Baseline	15	362.96	247.33		15	375.05	164.73		10	553.76	253.97	
Day 63	14	331.56	184.58	0.100	15	393.89	179.14	0.690	10	476.83	206.66	**0.001**
Day 180	11	415.97	316.04	0.403	10	407.37	181.43	0.225	10	574.80	276.30	**<0.001**
Day 300	8	415.49	296.70	0.728	10	379.20	255.24	0.131	3	571.18	62.30	**<0.001**
NfL (pg/ml)												
Baseline	15	5837.98	6210.15		15	301.65	203.87		10	449.58	403.76	
Day 63	14	2869.39	2463.28	0.127	15	216.33	89.22	0.114	10	342.21	236.26	0.374
Day 180	11	1551.40	3391.35	0.093	9	129.92	56.81	0.190	10	213.94	112.75	0.262
Day 300	7	361.96	236.89	**0.004**	10	133.94	85.19	0.199	3	192.01	126.53	0.613
pNF‐H (ng/ml)												
Baseline	15	1.78	2.19		15	0.19	0.13		10	0.32	0.27	
Day 63	14	0.27	0.18	**<0.001**	15	0.11	0.04	**0.002**	10	0.17	0.17	**0.046**
Day 180	11	0.17	0.15	**0.003**	9	0.08	0.04	**0.013**	10	0.10	0.08	**0.012**
Day 300	8	0.13	0.09	**0.005**	10	0.12	0.14	**0.029**	3	0.04	0.02	0.191

### 3.7 CSF proteins that have been investigated in a group of SMA patients before

#### 3.7.1 Neurofilaments (NfL, pNf‐H)

In all SMA types, mean NfL concentrations declined from baseline to day 300 with nusinersen treatment, but only in SMA type 1, this effect was significant. Initially, a significant increase in NfL concentrations was observed from baseline to treatment day 14 and 28 in SMA type 1 patients. No significant changes in concentrations were found in patients with SMA type 2 and type 3 and between the SMA groups (Table 4; Figure 3). Interestingly, baseline concentrations of NfL in patients with SMA type 2 and with SMA type 3 were about as high as the concentrations in SMA type 1 patients after 300 days of treatment, whereas baseline concentrations in SMA type 1 patients significantly differed compared to SMA type 2 and to type 3 patients.

pNf‐H concentrations decreased significantly with ongoing nusinersen treatment in SMA type 1 and 2 patients (Table 4; Figure 3). Data of only 3 SMA type 3 patients at treatment day 300 were available (Table 4) and should be interpreted with caution. No significant differences in baseline levels and concentration changes were observed between SMA types. According to the observations in NfL levels, mean concentrations after 2 months of treatment in SMA type 1 patients were lowered to those at baseline in SMA type 2 and 3 patients (Table 4; Figure 3).

### 3.8 CSF proteins that have not been investigated in a group of SMA patients before

#### 3.8.1 Total Tau (T‐Tau) protein

Highest baseline T‐Tau levels were found in SMA type 1 with lower levels in types 2 and 3, but baseline levels did not differ significantly between groups. In both SMA type 1 and 2 patients, T‐Tau levels significantly decreased from baseline to day 300 of treatment (Table 4; Figure 3).

### 3.9 Neurogranin

Significantly higher baseline levels were found in SMA type 3 compared to SMA type 2 and type 1. Only in SMA type 3, a significant increase in concentrations from baseline to treatment day 63, 180 and 300, respectively, were observed with significant group differences between SMA type 3 and the other SMA types (Table 4; Figure 3).

### 3.10 Alpha‐synuclein

In SMA type 1 patients, mean alpha‐synuclein levels increased on average with ongoing treatment. However, the age‐adjusted mean of concentration changes compared to baseline levels showed a significant decrease in further statistical analysis. Baseline levels were not significantly different between groups. In patients with SMA type 2/3, no significant effects regarding both mean concentration changes and concentration differences under treatment compared to baseline could be found (Table 4; Figure 3).

### 3.11 BACE‐1

At baseline, mean concentrations were lowest in SMA type 1 patients, we measured higher levels in SMA type 2 patients and highest levels in SMA type 3 patients. There were no significant differences in baseline concentrations between the SMA types. The course of BACE‐1 levels under nusinersen treatment in the different patient groups is shown in Figure 3. A relevant decrease in concentration (*p *= 0.056) was found from baseline to day 300 during treatment only in SMA type 1 (Table 4; Figure 3).

## 4 DISCUSSION

Spinal muscular atrophy is mainly associated with injury and loss of lower motor neurons, but there is also evidence for brain neuronal degeneration.^5^ The significance of this observation is unclear but of increasing importance considering new therapeutic options and an increasing life span of treated SMA patients. Different CSF proteins have been proposed to reflect CNS neurodegeneration and have been used as biomarkers in various neurological diseases mainly in adults but sporadically in children.^17^ In SMA patients, the spectrum of useful biomarkers for disease staging or monitoring disease progression is still under discussion. Therefore, we investigated a broad range of potential CSF proteins that might reflect the different proposed pathomechanisms in SMA and might serve as potential biomarkers in our pilot study. Overall, our data were most interesting for both neurofilaments and T‐Tau.

Neurofilaments are uniquely expressed in neurons, and neuronal injury and/or degeneration (as eg in amyotrophic lateral sclerosis (ALS)) lead to elevated neurofilament levels in CSF and plasma.^10^, ^18^, ^19^ In ALS patients, high concentrations have been found to correlate with increased disease severity and progression.^20^ The course of pNf‐H in plasma of nusinersen‐treated SMA type 1 patients was investigated in the ENDEAR treatment study.^15^ In the latter, higher baseline pNf‐H levels were associated with disease severity and a more rapid decline in pNf‐H levels was found in treated patients compared to those in the sham procedure control group. However, changes in motor function under nusinersen treatment were not reported. The initially elevated CSF pNf‐H levels and their rapid decline during treatment with nusinersen in our SMA type 1 patients are in line with the results in plasma for pNf‐H in the SMA type 1 patients of the ENDEAR study and the report of initially high NfL and pNf‐H levels and their decrease with nusinersen treatment in one SMA type 1 infant.^21^ Mean levels of pNf‐H at baseline in our SMA type 2 and 3 patients (0.19 and 0.32 ng/ml) were in or slightly above the normal range of healthy adults and were not as high as in patients with amyotrophic lateral sclerosis (see test instructions EUROIMMUN EQ 6561–9601 median levels in ALS patients: 2.12 ng/ml and in healthy controls: 0.17 ng/ml). Comparable values were observed in adult SMA type 3 patients^22^ and in some adolescent and adult SMA type 2 and 3 patients, respectively.^23^, ^24^ Baseline pNf‐H levels in SMA types 2 and 3 were similar to those after 2 months of treatment in SMA type 1 patients. Thus, pNf‐H might reflect both disease severity (as in SMA type 1) and activity and can be influenced by nusinersen treatment.

Changes in CSF NfL concentrations before and during nusinersen treatment in a group of SMA type 1 patients have not been reported yet. We observed decreasing NfL levels in all SMA groups after treatment onset, but significance was only reached in SMA type 1 patients. Shahim et al. reported similar median CSF levels of NfL in a group of 79 children aged 0.4–15.9 years without CNS disorders and much higher CSF NfL levels in children with different CNS disorders.^25^ In line with these data, average NfL baseline concentrations were markedly increased in our SMA type 1 patients compared to those in healthy children and in adults. However, the correlation between younger age and higher CSF NfL values was reported before and might contribute to our findings.^25^ Average baseline values in SMA type 2 and 3 patients were also elevated to levels slightly beyond those in healthy adults. However, in both, a group of adolescent and adult SMA type 2 and 3 patients^26^ and in 12 SMA type 3 patients,^22^ CSF NfL levels were similar to those in our patient group with SMA types 2 and 3. During nusinersen treatment, changes in CSF NfL levels were comparable to changes of CSF pNf‐H levels in our SMA patients. Applying this observation, NfL may be characterized as an additional marker for neuronal loss, mainly in SMA type 1 and possibly in paediatric SMA types 2 and 3.

We also addressed the question whether the course of neurofilaments in patients treated with nusinersen at day 63 and 180 could predict clinical treatment response and motor development. We observed changes in motor function scores under nusinersen treatment that reached significance for CHOP INTEND in SMA type 1 patients and for RULM in SMA type 2/3 patients. Also, there were significant changes of neurofilament concentrations in SMA type 1 patients (NfL and pNf‐H) and SMA type 2 patients (pNf‐H). However, the decrease of neurofilaments did not reach a statistical level of significance at day 63 and 180 as a predictive parameter. These results contradict the reported association between motor function and change in plasma pNf‐H levels in pre‐symptomatic SMA patients treated with nusinersen in the NURTURE study.^27^ Considering none of the patients included in our study were treated pre‐symptomatically, this is not surprising. However, patients undergoing nusinersen treatment changes in motor function scores correlated with changes of pNf‐H in all SMA subtypes and of NfL in SMA type 2/3 patients. In both NfL and p‐NfH, disease duration seems to be more relevant on baseline concentrations than ageing and/or maturational effects.

The microtubule‐associated Tau protein is involved in regulating intracellular trafficking and signal transduction and hyperphosphorylation of Tau is found in various neurodegenerative diseases (as eg Alzheimer's disease) but was also suggested to contribute to motor neuron degeneration in SMA.^28^ Increased CSF T‐Tau could be correlated with degeneration of cortical axons^29^, ^30^ and decreased CSF Tau levels were found during miglustat treatment in Niemann‐Pick type C patients, a therapy known to slow down disease progression.^31^ Recently, CSF T‐Tau levels below 200 pg/ml were found in 11 adult SMA type 3 patients before and during loading with nusinersen.^22^ However, in most of our SMA patients, baseline T‐Tau levels were above the normal range for adults (reference <300 pg/ml),^32^ median levels in healthy children^25^ and in children with leukaemia (244.84 ± 98.96 pg/ml).^33^ Tau levels dropped in all SMA patients during nusinersen treatment, but changes were only significant in SMA type 1 patients (after 2 months of treatment) and in SMA type 2 patients after 10 months of treatment. Statistical age adjustment should diminish the probability that the course of CSF T‐Tau levels in our SMA type 1 patients simply reflect ageing in infancy. Additionally, age‐dependent changes have previously been reported merely in the first 3 months of life.^34^ As baseline levels in our SMA patients were higher than normal, adult‐level T‐Tau protein might also reflect or contribute to the neuronal degeneration in early‐onset SMA. However, these findings have to be verified by comparing these data with a paediatric control group in additional studies. In SMA type 1, higher motor function scores significantly correlated with lower T‐Tau levels with nusinersen treatment making this parameter suitable as an outcome marker in severely affected patients.

Other investigated CSF proteins showed less encouraging results compared to neurofilaments and T‐Tau.

We chose to investigate the post‐synaptic protein neurogranin as its increase was linked to cognitive decline in patients with Alzheimer's disease possibly reflecting synaptic dysfunction and degeneration^35^, ^36^, ^37^ Cognitive function in children and adolescents with all SMA types has been reported within the normal range,^38^ but cognitive data of adult SMA patients or long‐term surviving SMA type 1 patients are missing or heterogeneous. Interestingly, neurogranin concentrations at baseline were above the normal adult values in our study (average levels: 180 pg/ml (range 125–273)^39^ and 159 pg/ml (92–105, 25.‐75. Quartiles^40^)). Furthermore, they increased in all SMA groups, however, only reaching a significant change during nusinersen treatment in SMA type 3 patients. Unfortunately, neurogranin values in healthy children are missing and interpretation of our results are limited by the lack of a paediatric control group. Therefore, the increase of neurogranin in our population has to be interpreted with caution in terms of cognitive function of SMA patients. Additionally, higher baseline concentrations might also reflect age‐appropriate levels in childhood.

After traumatic brain injury, elevated total alpha‐synuclein levels were found with values of about 4.09 ng/ml in adults and about 8.38 ng/ml in children,^41^, ^42^ suggesting widespread neurodegeneration and possibly synaptic dysfunction. This protein is mainly located presynaptically in cortical neurons,^43^ and its accumulation is also associated with neurodegeneration in synucleinopathies like Parkinson‘s disease.^44^ Median CSF levels of total alpha‐synuclein averaged around 1.32 ng/ml^41^ in healthy adults and around 0.463 ng/ml in healthy children aged 5 weeks to 14 years.^42^ In a study of Wennström et al.,^45^ CSF alpha‐synuclein levels were low in patients with synucleinopathies compared to patients with Alzheimer's disease or the healthy control group. In our SMA patients, concentrations were not as high as those after traumatic brain injury but higher than those in a small group of healthy children^42^ possibly indicating affection of cortical neurons. However, our data are difficult to interpret as normal CSF alpha‐synuclein concentrations in a larger group of infants and children are not yet available. Concentration changes of alpha‐synuclein at day 180 correlated with improvement in motor function in SMA type 1 patients at day 300. This finding has to be kept in mind in future evaluations of this CSF protein in SMA but we would be cautious to consider it as a predictive biomarker of clinical outcome at this stage.

Finally, we investigated the CSF ß‐secretase 1 (BACE‐1) as an increase of the protein has been suggested to reflect the intensity of axonal degeneration in adult patients with Alzheimer's disease.^46^ Although we found a similar correlation between clinical outcome and course of BACE‐1 concentrations after 6 months of treatment as aforementioned for alpha‐synuclein, our overall data regarding BACE‐1 provide no convincing evidence that this protein might reflect pathogenic mechanisms important for SMA.

In conclusion, our findings suggest that pNf‐H and NfL are correlated with disease severity and activity pronouncing their relevance as marker of neuronal loss and clinical outcome. T‐Tau was significantly correlated with motor function scores in SMA type 1 making it an interesting marker for treatment response. Additionally, T‐Tau might also reflect the extension of neuronal degeneration in paediatric‐onset SMA. The results of the other CSF proteins are interesting but certainly have to be interpreted with caution because of our small sample cohort and missing normal values in healthy children of different ages. We minimize this issue by focusing on intraindividual changes of the parameters and considering age adjustment in the statistics thereby addressing ageing effects within our study population. As nusinersen is supposed to slow down but not to stop disease progression, we cannot fully exclude that the latter leads to a decreasing amount of motor neurons contributing to the decline of some CSF proteins (NfL, p‐NfH, T‐Tau) over time but increasing motor function scores in our cohort contradicts this consideration. We acknowledge the limitations of our data, due to the small sample size in our pilot study, especially in the group of SMA type 3 patients after 10 months of treatment. Moreover, lacking a paediatric control group provides another limitation considering we had to refer to normal values mentioned in the literature for the CSF proteins NfL, T‐Tau and alpha‐synuclein. Additionally, higher baseline levels of the investigated CSF proteins in SMA type I patients in the youngest age group might reflect an age‐appropriate finding. Therefore, our data should be interpreted as explorative results of a single centre to encourage additional studies.

## CONFLICTS OF INTEREST

The authors DW, LS, AD and JD declare that they have no competing interests. JJ received advisory board and/or speaker honoraria and financial support for conference attendances from Avexis, Biogen, Roche, PTC and Sarepta.

## AUTHOR CONTRIBUTIONS

**Jessika Johannsen:** Conceptualization (lead); Formal analysis (lead); Project administration (lead); Writing‐original draft (lead). **Deike Weiss:** Project administration (equal); Resources (equal); Writing‐review & editing (equal). **Anne Daubmann:** Formal analysis (lead); Writing‐review & editing (equal). **Leonie Schmitz:** Formal analysis (equal). **Jonas Denecke:** Conceptualization (lead); Formal analysis (lead); Writing‐original draft (lead).

## Data Availability

The data sets used and/or analysed during the current study are available from the corresponding author on reasonable request.

## References

[1] http://www.ema.europa.eu/en/medicines/human/EPAR/spinraza

[2] Monani UR, Lorson CL, Parsons DW, et al. A single nucleotide difference that alters splicing patterns distinguishes the SMA gene SMN1 from the copy gene SMN2. Hum Mol Genet. 1999;8:1177‐1183 10369862 10.1093/hmg/8.7.1177

[3] Hua Y, Sahashi K, Rigo F, et al. Peripheral SMN restoration is essential for long‐term rescue of a severe spinal muscular atrophy mouse model. Nature. 2011;478:123‐126 21979052 10.1038/nature10485 PMC3191865

[4] Wirth B, Brichta L, Schrank B, et al. Mildly affected patients with spinal muscular atrophy are partially protected by an increased SMN2 copy number. Hum Genet. 2006;119:422‐428 16508748 10.1007/s00439-006-0156-7

[5] Harding BN, Kariya S, Monani UR, et al. Spectrum of neuropathophysiology in spinal muscular atrophy type I. J Neuropathol Exp Neurol. 2015;74:15‐24 25470343 10.1097/NEN.0000000000000144 PMC4350580

[6] Michelson D, Ciafaloni E, Ashwal S, et al. Evidence in focus: Nusinersen use in spinal muscular atrophy: Report of the Guideline Development, Dissemination, and Implementation Subcommittee of the American Academy of Neurology. Neurology. 2018;91:923‐933 30315070 10.1212/WNL.0000000000006502

[7] Finkel RS, Mercuri E, Darras BT, et al. Nusinersen versus Sham Control in Infantile‐Onset Spinal Muscular Atrophy. N Engl J Med. 2017;377:1723‐1732 29091570 10.1056/NEJMoa1702752

[8] Mercuri E, Darras BT, Chiriboga CA, et al. Nusinersen versus Sham Control in Later‐Onset Spinal Muscular Atrophy. N Engl J Med. 2018;378:625‐635 29443664 10.1056/NEJMoa1710504

[9] Mendell JR, Shell L, Lehman KJ, et al. Gene‐Replacement Therapy (GRT) in Spinal Muscular Atrophy Type 1 (SMA1): Long‐Term Follow‐Up from the Onasemnogene Abeparvovec Phase 1/2 Clinical Trial. J Neurol Sci. 2019;. 10.1016/j.jns.2019.10.1322.oi.org/10.1016/j.jns.2019.10.1322

[10] Rosengren LE, Karlsson JE, Karlsson JO, et al. Patient with ALS and other neurodegenerative disease have increased levels of neurofilament protein. J Neurochem. 1996;67:2013‐2018 8863508 10.1046/j.1471-4159.1996.67052013.x

[11] Willem M, Garratt AN, Novak B, et al. Control of peripheral nerve myelination by the ß‐secretase BACE1. Science. 2006;314:664‐666 16990514 10.1126/science.1132341

[12] Kolarcik C, Bowser R. Plasma and cerebrospinal fluid‐based protein biomarkers for motor neuron disease. Mol Diagn Ther. 2006;10(5):281‐292 17022691 10.1007/BF03256203

[13] Brück D, Wenning GK, Stefanova N, et al. Glia and alpha‐synuclein in neurodegeneration: a complex interaction. Neurobiol Dis. 2016;85:262‐274 25766679 10.1016/j.nbd.2015.03.003 PMC4730552

[14] Kolb SJ, Coffey CS, Yankey JW, et al. Baseline results of the Neuro NEXT spinal muscular atrophy infant biomarker study. Ann Clin Transl Neurol. 2016;3(2):132‐145 26900585 10.1002/acn3.283 PMC4748311

[15] Darras BT, Crawford TO, Finkel RS, et al. Neurofilament as a potential biomarker for spinal muscular atrophy. Ann Clin Transl Neurol. 2019;6(5):932‐944 31139691 10.1002/acn3.779 PMC6530526

[16] Fadda G, Kichula E, Bacchus M, et al. CSF Biomarkers of Disease Severity and Response to Nusinersen Treatment in Children with Spinal Muscular Atrophy. Neurology. 2020;94:1971

[17] Shahim P, Månsson JE, Darin N, et al. Cerebrospinal fluid biomarkers in neurological diseases in children. Eur J Paediatr Neurol. 2013;17(1):7‐13 23026858 10.1016/j.ejpn.2012.09.005

[18] De Schaepdryver M, Goossens J, De Meyer S, et al. Serum neurofilament heavy chains as early marker of motor neuron degeneration. Ann Clin Transl Neurol. 2019;6(10):1971‐1979 31518073 10.1002/acn3.50890 PMC6801162

[19] De Schaepdryver M, Jeromin A, Gille B, et al. Comparison of elevated phosphorylated neurofilament heavy chains in serum and cerebrospinal fluid of patients with amyotrophic lateral sclerosis. J Neurol Neurosurg Psychiatry. 2018;89(4):367‐373 29054919 10.1136/jnnp-2017-316605

[20] Tortelli R, Ruggieri M, Cortese R, et al. Elevated CSF neurofilament light levels in patients with ALS: a possible marker of disease severity and progression. Eur J Neurol. 2012;9(12):1561‐1567 10.1111/j.1468-1331.2012.03777.x 22680408

[21] Winter B, Guenther R, Ludolph AC, et al. Neurofilaments and tau in CSF in an infant with SMA type 1 treated with nusinersen. J Neurol Neurosurg Psychiatry. 2019;90(9):1068‐1069 10.1136/jnnp-2018-320033 30630960

[22] Totzeck A, Stolte B, Kizina K, et al. Neurofilament Heavy Chain and Tau Protein Are Not Elevated in Cerebrospinal Fluid of Adult Patients with Spinal Muscular Atrophy during Loading with Nusinersen. Int J Mol Sci. 2019;20(21):5397 10.3390/ijms20215397 PMC6862027 31671515

[23] Wurster CD, Günther R, Steinacker P, et al. Neurochemical markers in CSF of adolescent and adult SMA patients undergoing nusinersen treatment. Ther Adv Neurol Disord. 2019;10(12):1756286419846058 10.1177/1756286419846058 PMC6535708 31205491

[24] Faravelli I, Meneri M, Saccomanno D, et al. Nusinersen treatment and cerebrospinal fluid neurofilaments: An explorative study on Spinal Muscular Atrophy type 3 patients. J Cell Mol Med. 2020;24(5):3034‐3039 32032473 10.1111/jcmm.14939 PMC7077557

[25] Shahim P, Darin N, Andreasson U, et al. Cerebrospinal fluid brain injury biomarkers in children: a multicenter study. Pediatr Neurol. 2013;49(1):31‐39 23827424 10.1016/j.pediatrneurol.2013.02.015

[26] Wurster CD, Steinacker P, Günther R, et al. Neurofilament light chain in serum of adolescent and adult SMA patients under treatment with nusinersen. J Neurol. 2020;267(1):36‐44 10.1007/s00415-019-09547-y 31552549

[27] Muntoni F, Sumner C, Darras B, et al. Association between plasma phosphorylated neurofilament heavy chain and efficacy endpoints in the nusinersen NURTURE study. Neuromuscul Dis. 2019;29:S146

[28] Miller N, Feng Z, Edens BM, et al. Non‐aggregating tau phosphorylation by cyclin‐dependent kinase 5 contributes to motor neuron degeneration in spinal muscular atrophy. J Neurosci. 2015;35(15):6038‐6050 25878277 10.1523/JNEUROSCI.3716-14.2015 PMC4397602

[29] Kay GW, Verbeek MM, Furlong JM, et al. Neuropeptide changes and neuroactive amino acids in CSF from humans and sheep with neuronal ceroid lipofuscinoses (NCLs, Batten disease). Neurochem Int. 2009;55(8):783‐788 19664668 10.1016/j.neuint.2009.07.012 PMC2764820

[30] Mattsson N, Zetterberg H, Bianconi S, et al. γ‐Secretase‐dependent amyloid‐β is increased in Niemann‐Pick type C: A cross‐sectional study. Neurology. 2011;76(4):366‐372 21205675 10.1212/WNL.0b013e318208f4ab PMC3034414

[31] Mattsson N, Zetterberg H, Bianconi S, et al. Miglustat treatment may reduce cerebrospinal fluid levels of the axonal degeneration marker tau in Niemann‐Pick type C. JIMD Rep. 2012;3:45‐52 23430872 10.1007/8904_2011_47 PMC3509856

[32] Sjögren M, Vanderstichele H, Ågren H, et al. Tau and Aβ42 in cerebrospinal fluid from healthy adults 21–93 years of age: establishment of reference values. Clin Chem. 2001;47(10):1776‐1781 11568086

[33] Muszyńska‐Rosłan K, Krawczuk‐Rybak M, Protas PT, et al. Level of tau protein in children treated for acute lymphoblastic leukemia. Pediatr Neurol. 2006;34(5):367‐371 16647996 10.1016/j.pediatrneurol.2005.10.018

[34] Lofberg H, Grubb AO, Sveger T, Olsson JE. The cerebrospinal fluid and plasma concentrations of gamma‐trace and beta2‐microglobulin at various ages and in neurological disorders. J Neurol. 1980;223:159‐170 6157009 10.1007/BF00313180

[35] Huang KP, Huang FL, Jäger T, et al. Neurogranin/RC3 enhances long‐term potentiation and learning by promoting calcium‐mediated signaling. J Neurosci. 2004;24(47):10660‐10669 15564582 10.1523/JNEUROSCI.2213-04.2004 PMC6730132

[36] De Vos A, Jacobs D, Struyfs H, et al. C‐terminal neurogranin is increased in cerebrospinal fluid but unchanged in plasma in Alzheimer's disease. Alzheimers Dement (N Y). 2015;11(12):1461‐1469 10.1016/j.jalz.2015.05.012 26092348

[37] Kvartsberg H, Duits FH, Ingelsson M, et al. Cerebrospinal fluid levels of the synaptic protein neurogranin correlates with cognitive decline in prodromal Alzheimer's disease. Alzheimers Dement (N Y). 2015;11(10):1180‐1190 10.1016/j.jalz.2014.10.009 25533203

[38] Von Gontard A, Zerres K, Backes M, et al. Intelligence and cognitive function in children and adolescents with spinal muscular atrophy. Neuromuscul Dis. 2002;12(2):130‐136 10.1016/s0960-8966(01)00274-7 11738354

[39] Lista S, Toschi N, Baldacci F, et al. Cerebrospinal fluid neurogranin as a biomarker of neurodegenerative diseases: a cross‐sectional study. J Alzheimers Dis. 2017;59(4):1327‐1334 28731449 10.3233/JAD-170368

[40] De Vos A, Struyfs H, Jacobs D, et al. The cerebrospinal fluid neuograinin/BACE1 is a potential correlate of cognitive decline in alzheimer’s disease. J Alzheimers Dis. 2016;53(4):1523‐1538 27392859 10.3233/JAD-160227 PMC4981899

[41] Mondello S, Buki A, Italiano D, et al. α‐Synuclein in CSF of patients with severe traumatic brain injury. Neurology. 2013;80(18):1662‐1668 23553480 10.1212/WNL.0b013e3182904d43

[42] Su E, Bell MJ, Wisniewski SR, et al. α‐Synuclein levels are elevated in cerebrospinal fluid following traumatic brain injury in infants and children: the effect of therapeutic hypothermia. Dev Neurosci. 2010;32(5–6):385‐395 21124000 10.1159/000321342 PMC3073758

[43] Norris EH, Giasson BI, Lee VMY. α‐Synuclein: normal function and role in neurodegenerative diseases. Current topics in developmental biology. Academic Press; 2004:17‐54 10.1016/S0070-2153(04)60002-0 15094295

[44] Fujiwara H, Hasegawa M, Dohmae N, et al. α‐Synuclein is phosphorylated in synucleinopathy lesions. Nat Cell Biol. 2002;4(2):160 11813001 10.1038/ncb748

[45] Wennström M, Surova Y, Hall S, et al. Low CSF levels of both α‐synuclein and the α‐synuclein cleaving enzyme neurosin in patients with synucleinopathy. PLoS One. 2013;8(1):e53250 23308173 10.1371/journal.pone.0053250 PMC3540093

[46] Zetterberg H, Andreasson U, Hansson O, et al. Elevated cerebrospinal fluid BACE1 activity in incipient Alzheimer disease. Arch Neurol. 2008;65(8):1102‐1107 18695061 10.1001/archneur.65.8.1102

